# O.2.1-9 Are doctors and nurses engaging in physical activity and its promotion?

**DOI:** 10.1093/eurpub/ckad133.114

**Published:** 2023-09-11

**Authors:** Samantha Fawkner, Sara Bhandari, Emma Watkinson

**Affiliations:** University of Edinburgh, UK; s.fawkner@ed.ac.uk; University of Edinburgh, UK; s.fawkner@ed.ac.uk; University of Edinburgh, UK; s.fawkner@ed.ac.uk

## Abstract

**Purpose:**

Healthcare professionals (HCPs) are ideal providers of physical activity (PA) promotion; a large portion of the population have contact with a form of HCP and they are viewed as trusted and respected role models for health advice. Despite this, we have a very limited understanding of HCPs PA counselling practices. The aim of this study was to evaluate PA levels, attitudes towards PA counselling and counselling practices of UK doctors and nurses.

**Methods:**

The study included two cross-sectional, anonymised, online surveys conducted in 2018/19 (survey 1) and 2019/20 (survey 2). Whether or not participants were achieving the aerobic PA guideline was evaluated using the first 2 questions of the Scot-PASQ survey. Counselling practices were assessed using likert scales to assess the proportion of patients that participants had discussed PA with in the past week and their perceived importance of PA counselling as part of their role.

**Results:**

460 doctors and 169 nurses from the UK completed the survey. 78.3% of doctors and 73.4% of nurses met the UK aerobic PA guidelines. Perceived importance of counselling was high but participants were providing PA counselling on average for less than 25% of their patients. PA counselling was more likely in primary care and doctors were marginally more likely than nurses to counsel on PA.

**Conclusion:**

This cohort of doctors and nurses were active and viewed PA counselling as important. Despite this, PA counselling levels were low especially in secondary care. Efforts should be made to improve knowledge of, and opportunity for, PA counselling in HCPs.

